# Environmentally Sustainable Achiral and Chiral Chromatographic Analysis of Amino Acids in Food Supplements

**DOI:** 10.3390/molecules27227724

**Published:** 2022-11-09

**Authors:** Ina Varfaj, Andrea Carotti, Luciano Mangiapelo, Lina Cossignani, Agnese Taticchi, Antonio Macchiarulo, Federica Ianni, Roccaldo Sardella

**Affiliations:** 1 Department of Pharmaceutical Sciences, University of Perugia, Via Fabretti 48, 06123 Perugia, Italy; 2 Center for Perinatal and Reproductive Medicine, University of Perugia, Santa Maria della Misericordia University Hospital, 06132 Perugia, Italy; 3 Department of Agricultural Food and Environmental Sciences, University of Perugia, Via S. Costanzo, 06126 Perugia, Italy

**Keywords:** enantiorecognition mechanism, green chromatography, ion-pairing agent, teicoplanin-based stationary phase

## Abstract

Two LC methods were developed for the achiral and chiral reversed-phase (RP) analysis of an amino acid (AA) pool in a food supplement, in compliance with the main paradigms of Green Chromatography. A direct achiral ion-pairing RP-HPLC method was optimized under gradient conditions with a water-ethanol (EtOH) eluent containing heptafluorobutyric acid (0.1%, *v/v*), to quantify the eight essential AAs (Ile, Leu, Lys, Met, Phe, Thr, Trp, and Val) contained in the food supplement. Thus, the usually employed acetonitrile was profitably substituted with the less toxic and more benign EtOH. The method was validated for Leu and Phe. The chiral LC method performed with a teicoplanin chiral stationary phase was developed with a water-EtOH (60:40, *v/v*) eluent with 0.1%, *v/v* acetic acid. The enantioselective analysis was carried out without any prior derivatization step. Both developed methods performed highly for all eight AAs and revealed that: (i) the content of six out of eight AAs was consistent with the manufacturer declaration; (ii) only L-AAs were present. Furthermore, it was demonstrated that a two-dimensional achiral–chiral configuration is possible in practice, making it even more environmentally sustainable. A molecular modelling investigation revealed interesting insights into the enantiorecognition mechanism of Lys.

## 1. Introduction

Amino acids (AAs) are building blocks for tissue proteins and essential substrates for the synthesis of many low molecular-weight substances involved in important physiological processes. Among AAs, “Essential AAs (EAAs)” are defined as those nine AAs that either cannot be synthesized de novo by the body or are synthesized in quantities too small to supply the body needs. Therefore, EAAs must be supplied with the diet to meet optimal requirements [1]. EAAs are the following species: phenylalanine (Phe), valine (Val), tryptophan (Trp), threonine (Thr), isoleucine (Ile), methionine (Met), histidine (His), leucine (Leu), and lysine (Lys) [1]. Over the last two decades, the new concept of “Functional AAs (FAAs)” has emerged indicating the EAAs that mostly contribute to regulate key metabolic pathways responsible for the improvement of the health state as a whole and that of the reproductive systems, as well as of the growth, development, and lactation. FAAs comprise arginine (Arg), cysteine (Cys), His, Leu, Met, Trp, and tyrosine (Tyr).

A body of evidence strongly suggests that some EAAs (mostly, Thr, Val, Lys, Met, Ile, Leu, Phe, and Trp) are often scarcely included in the diet, which justifies the considerable increase in the consumption of AAs-based food supplements in recent years [2,3]. Food supplements, also known as dietary supplements, are concentrated sources of nutrients or other substances producing one or more beneficial nutritional or physiological effects, which can be prescribed in many alternative ways including capsules, tablets, pills, sachets of powder, etc. [4,5,6].

In spite of the great diffusion of food supplements worldwide, their market is however still highly vulnerable and often “polluted” by intentional alterations with other substances [7]. In this scenario, it is very surprising that practically no country has a strict legislation regarding both the quality control (purity assessment) of the standards used for food supplement ingredients and the protection of consumers against commercial frauds. It is indeed well-known that the overall safety of a dietary supplement is greatly affected by the purity of the functional ingredients, and there are serious adverse effects derived from either the unintentional or the deliberate addition (economically motivated) of other active substances. As far as the AAs containing food supplements are concerned, specific attention should be paid to the control of stereochemistry. All commercialized food supplements indicate the exclusive presence of L-AAs, while implicitly excluding the presence of their D isomers. Nowadays, it is fully acknowledged that D-AAs play important physiological roles in many biological systems, which can be different from those activated by their antipodes [8,9]. This evidence implies the obvious necessity to ascertain the enantiomeric composition of the individual AA present in a food supplement since different “pharmaco-toxicological” profiles besides nutritional properties are stereochemically-related issues. In most of the cases, D-AAs can either be converted to L-AAs, thus contributing to the total L-AAs content or act similarly to L-AAs in a specific process but at a different rate [8,10,11]. However, it has been also documented that D-AAs can both act stereo-specifically independently from L-AAs and even act antagonistically to L-AAs.

All the above concerns clearly highlight the importance of making analytical methods available for the purpose of quality control of food supplements, including the accurate determination of the enantiomeric composition of chiral ingredients. Nowadays, to meet the great ambitions of the “Global Green New Deal”, among the multiple actions undertaken by the pharmaceutical industry, the progressive shift towards environmentally sustainable analytical methods is of great importance. Due to the large amount of analysis required in most of the production pipelines of food supplements, this trend would be auspicial also in the food and food supplements industries. Among the main objectives of the “Green Analytical Chemistry (GAC)” and, more specifically, of the “Green Chromatography (GC)”, the reduction (or even the elimination) of highly toxic organic solvents and other toxic reagents in the analytical procedures, the automation of the analytical procedures, and the reduction of waste and reuse of solvents and materials are worth highlighting [12]. Conventional liquid chromatography (LC) methods performed both under normal- and reversed-phase (NP and RP, respectively) conditions, usually require the use of large quantities of highly toxic and volatile organic solvents. In this context, the replacement of conventional organic solvents with less toxic alternatives is of particular relevance in the design of new sustainable analytical methods [13,14]. For example, organic solvents such as acetonitrile (ACN) and hydrocarbons are flammable, toxic, and characterized by elevated costs from purchase to disposal, thereby stimulating their substitution with other valuable no more convenient alternatives. An important issue related to the implementation of GC principles is however the need to adequately balance the environmental friendliness of the method with its statistical quality, which sometimes contradict each other.

The main aim of this study was the development and application of environmentally friendly and mass-spectrometry (MS) fully compatible LC methods for the achiral and chiral analysis of an AAs pool in a selected commercial food supplement labelled to contain eight out of nine of the EAAs, that is, Phe, Val, Trp, Thr, Ile, Met, Leu, and Lys. With this study, we want to produce further evidence that accurate analyses of these eight AAs, which are present in a multitude of commercial food supplements, can be conveniently performed even without the need of prior derivatization processes. Furthermore, herein, we also aim to demonstrate that the use of the “benign solvent” ethanol (EtOH) in water/EtOH-based mobile phases [15] does not impair the statistical quality of the methods, compared to those usually applied for the same purpose. More to the point, we demonstrate that the two methods (achiral and chiral) can be profitably combined in a two-dimensional HPLC (2D-HPLC) heart-cut configuration, making the developed methods even more environmentally sustainable. Finally, we produced evidence that molecular modelling investigations can contribute to the understanding of the basic mechanisms underlying the enantiorecognition processes, thereby making possible the reduction of non-useful chromatographic analyses, in strict accordance with the main paradigms of GC [12,13,14,16].

## 2. Results and Discussion

As already anticipated in the Introduction, the main objective of this work was the development of combined direct HPLC achiral and chiral methods enabling the overall analysis of specific AAs contained in a test food supplement in compliance with the main principles of GC [17]. With this aim, we focused our attention on the exclusive selection of harmless and environmentally friendly solvents and mobile phases components.

In the following sections, the development of the methods for the achiral and chiral analysis is described in detail.

### 2.1. Development of the Achiral IP-RP-HPLC Method

Based on several successful studies on the analysis of AA mixtures in food matrices carried out both by other groups [18,19] and in our laboratory [20,21], we decided to develop an ion-pair reversed-phase (IP-RP) HPLC method by replacing the commonly used ACN with the greener and less toxic EtOH.

Owing to its low viscosity, complete miscibility with water and low UV absorption, ACN is still the most widespread organic modifier for RP-HPLC applications, also when used in combination with IP reagents. However, in spite of all these favorable characteristics, its well-recognized acute toxicity (both dermal and oral), flammability, and volatility, besides its inclusion in the list of hazardous air pollutants [22], has stimulated many researchers worldwide to find valuable alternatives in compliance with the main paradigms of sustainable development. This tendency was further fueled by the global shortage of ACN in 2009 [23]. Compared to ACN, EtOH exhibits lower toxicity, higher facility of disposal, reduced cost, and the same or even superior characteristics for the HPLC separation of low, middle, and high-molecular weight biomolecules under IP-RP conditions [24]. All these reasons have collectively contributed to classify EtOH as a benign solvent with favorable environmental, health, and safety indicators [15]. Nevertheless, to the best of our knowledge, the IP-RP-HPLC analysis of a pool of underivatized proteinogenic AAs with EtOH as the organic modifier in the aqueous mobile phase is still missing in the literature.

In the present study, HFBA was selected as volatile additive in the mobile phase, for its well-known ability in increasing retention of compounds carrying amino groups, as a result of the enhanced lipophilicity of the formed ion-pair. In many instances, the more favorable retention behavior was accompanied by an improved chromatographic selectivity and elution efficiency [18,19,20,21]. Although perfluorinated carboxylic acids with longer side chains have been reported to produce higher chromatographic performances than HFBA, this short-chain additive represents the best compromise holding the advantage to avoid prolonged re-equilibration times between consecutive runs under gradient elution conditions, which are very common with other long-chain perfluoroalkylic acids [25].

An eluent system composed of water/EtOH/HFBA in the ratio 80:20:0.1 (*v/v/v*) was initially tested under isocratic conditions with an artificial mixture made up of the eight AAs contained in the food supplement. In accordance with literature data, the amino acids Thr, Val, Met, *allo*-Il/Ile, Leu, Phe, and Trp were eluted in direct relation to their lipophilicity (Table 1) [20,21]. Instead, the singular behavior displayed by Lys was the result of multiple factors including the analyte charge and polarity, as well as the hydrophobicity of the produced ion-pair and its relative interactions with the octadecyl chains of the stationary phase. Noteworthy, the elution order in the EtOH-based system was exactly the same previously obtained by our group with water-ACN mobile phases [20,21], even though, the alcoholic modifier produced lower elution efficiencies than ACN, as a result of the slower mass transfer kinetics [20,21]. In order to obtain the baseline separation of the couple Lys/Val (which represented the unique critical resolution evidenced by this experimental setting), the effect of an increase in the HFBA content (0.2%, *v/v*) was tested. Consistent with the IP-RP retention mechanism [26], an increase in retention was observed for almost all compounds (the unique exception being Trp), with the co-elution of Lys and Val. The separation between other consecutive peaks remained almost unchanged and was only little affected by this experimental modification.

In order to further improve the thermodynamic and kinetic characteristics of the IP-RP-HPLC system, lower percentages of EtOH in the eluent were progressively scrutinized under isocratic elution conditions, keeping the HFBA content fixed at 0.1% (*v/v*). In accordance with an RP mechanism, an increase in the water content was paralleled by an increase in the residence time of the investigated analytes into the column, with a favorable improvement of the separation (α) and resolution (R_S_) factor values (Table 1). Apart from the excessive retention of Phe and Trp, excellent results were obtained for the remaining six compounds when the EtOH content was reduced down to 5% (*v/v*) (Table 1).

**Figure 1:**
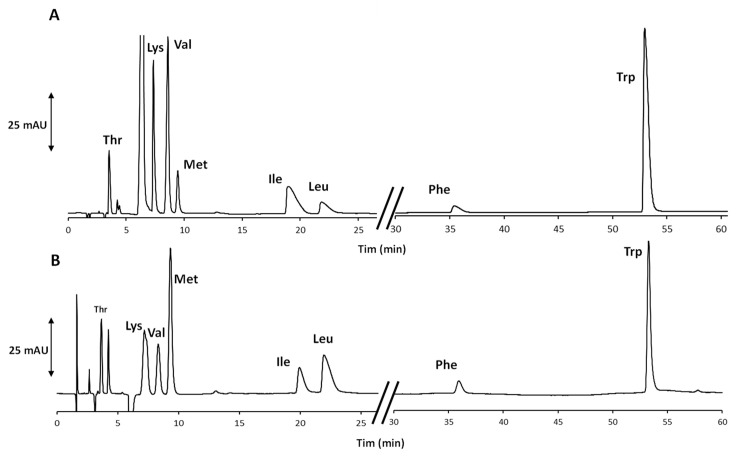
Chromatograms of (**A**) the mixture of eight AA standards and (**B**) the extract from the selected food supplements. The chromatographic conditions correspond to those labelled as “f” in the footnote to Table 1.

The unprofitable retention behavior of Phe and Trp was easily overcome by progressively increasing the EtOH content in the eluent after the elution of Leu. More details about the optimized gradient program are reported in Section 2.3 and in the footnote to Table 1. In this way, both aromatic compounds were eluted in less than 70 min, and an even better result was achieved increasing the eluent flow rate from 1.0 up to 1.2 mL/min, applying the optimized combination of the isocratic and gradient elution modes. As clearly evident from the chromatogram in Figure 1A related to the analysis of an artificial AA standards mixture, the increase in the eluent flow rate did not impair the chemoselectivity of the system and had a positive effect on the peak shapes. Based on the eluent composition, the method was in evident adherence to the main principles of GC [17], and therefore, it was applied to the analysis of the real sample. In Figure 1B, the chromatogram of the extract is shown.

A substantial reduction of the retention time is made possible in principle by the use of a (not so widely diffused) U(H)PLC system allowing to work with a column of extremely small particle size (<2 μm). However, one of the main objectives of GC is to develop methods which can be exploited even in laboratories located in emerging countries (emerging economies) where budget constraints often preclude analytical chemists from accessing very expensive instrumentations [27,28,29]. For this reason, a method operated with conventional HPLC systems has been here preferred. However, in order to demonstrate that shorter analysis times can be obtained with HPLC columns of reduced dimension, a method allowing the analysis of the eight AAs in about 20 min was developed. The result of this additional analysis has been reported as Supplementary Information (Figure S1).

### 2.2. Validation of the Optimized Achiral IP-RP-HPLC Method and Quantitative Analysis of the AAs in the Food Supplement

A “research (partial) validation” study of the developed IP-RP-HPLC method was performed on only two AAs, Leu and Phe, which were arbitrarily selected as representative of aliphatic and aromatic species withing the food supplement. Moreover, Leu and Phe were analyzed under different conditions, isocratic and under gradient elution, respectively. The following parameters were considered in the validation study: specificity, linearity, precision (in the short- and long-period), accuracy (in the short- and long-period), and limit of quantification (LOQ).

Method specificity was evaluated by injecting 20 μL of blank (mobile phase). No system peak was detected at the retention time of neither Leu nor Phe.

To appraise the accuracy and precision of the method, an external set of two control solutions was selected both for Leu (at the 0.11 and 0.45 mg/mL concentration levels) and Phe (at the 0.02 and 0.16 mg/mL concentration levels). These solutions were analyzed in triplicate on the same day and for three different days (Table 2). The calibration curves previously defined for Leu and Phe were then applied to calculate the concentration of each selected control solution.

The system precision was determined as the relative standard deviation (RSD%) among the concentration values achieved from consecutive injections, respectively, within the short- or the long-term period. The same experimental protocol was applied to assess the method accuracy expressed as Recovery%.

As evident from Table 2, high Recovery% values for Leu (100.1% and 99.6% for the two external set solutions, respectively) and Phe (98.3% and 100.6% for the two external set solutions, respectively) were found in the long-term (inter-day) accuracy. Additionally, low RSD% values for Leu (1.9% and 1.5%, for the two external set solutions, respectively) and Phe (1.8% and 1.5%, for the two external set solutions, respectively) turned out as well. In order to further estimate the Recovery% of the method, extracts were also spiked with standard solutions of either L-Leu or L-Phe at two different concentration levels. For both compounds, the theoretical concentration levels were established in a way to be included within the range of area values obtained analyzing the five selected calibration standard solutions: 0.30 mg/mL and 0.43 mg/mL for L-Leu, and 0.08 mg/mL and 0.14 mg/mL for L-Phe. All the four solutions were run in triplicate. The recovery% was evaluated using the calibration curves previously built-up for the two AAs, as follows: (C_experimental_)/(C_theoretical_) × 100. As a result, mean recovery% values of 96.2% and 103.4% were measured for L-Leu and L-Phe, respectively.

The LOQ was assessed considering the minimum concentration at which the RSD% of the peak area values from five consecutive injections was higher than 5.0%. Accordingly, LOQ values of 0.03 mg/mL and 0.006 mg/mL were established for Leu and Phe, respectively.

The validation study performed on Leu and Phe allowed us to ascertain the high statistical quality of the developed method, which was therefore applied for the quantitative analysis of the single AAs contained in the selected food supplement. Extract solutions were analyzed in triplicate with the optimized and validated IP-RP-HPLC method. An excellent repeatability among successive runs was obtained both for retention times and peak area values. Indeed, the RSD%s for the former were ≤ 0.22, while for the latter ≤ 2.50. The quantitative analysis of the food supplement revealed that six out of eight AAs were present in amount very close (from approximately 90% up to 95%) to that declared by the producer, while it was lower for Met (close to 80%) and Trp (close to 70%).

As the developed method was able to distinguish Ile from *allo*-Ile, the presence of the latter was excluded in the food supplement. *Allo*-Ile could indeed be formed as by-product of its diastereomer Ile during technological processing [30].

The relatively lower amounts found for Met and Trp can be ascribed to multiple aspects. In a recent review paper, Bellmaine and co-workers [31] reported that Trp is prone to undergo oxidative degradation as a consequence of light exposure, presence of reactive oxygen species and carbonyl-containing organic molecules, as well as pH changes, increased temperature, and metal cations exposure. An even more pronounced attitude to oxidation is well-documented for Met, due the presence of a thioether group in its sidechain [32]. Met is one of the most oxidation-prone AAs, and its low chemical and thermal stability represents a major challenging degradation pathway of proteins in food and therapeutics. The degradation process affecting these two AAs could plausibly occur during the manufacturing/formulation process even though some effects by the analytical method (including the extraction from the food supplement and the chromatographic analysis) cannot be ruled out. However, the unexpected result for Met and Trp did not derive from an incomplete extraction from the formulation, since multiple water-EtOH solutions of different polarity were tested leading to comparable results.

### 2.3. Development of the Enantioselective Liquid-Chromatographic Method and Application to the Real Sample

Different valuable HPLC methods have been developed for the direct enantioseparation of proteinogenic AAs [32,33,34,35,36]. However, only a few of these are environmentally sustainable and MS compatible at once. The direct enantioseparation of AAs with a teicoplanin-based CSP was firstly described by Berthod and co-workers in 1996 [37]. In that paper, the authors reported a very interesting number of examples highlighting the ability of the glycopeptide in separating, inter alia, the enantiomers of all 20 proteinogenic AAs under RP conditions and green water-alcohol containing eluent systems fully compatible with MS detectors.

**Figure 2:**
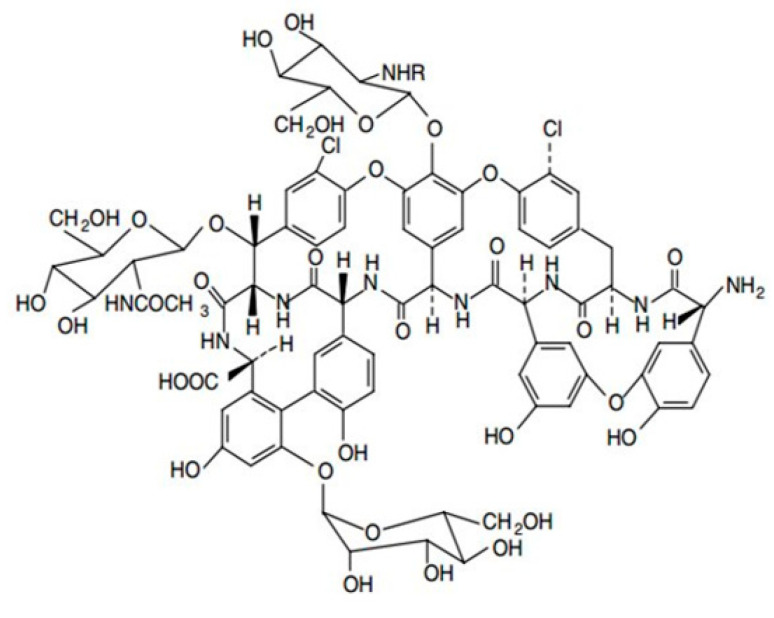
Structure of the teicoplanin-based chiral selector.

From a structural point of view, the aglycone basket of the teicoplanin chiral selector contains a single primary amine (pK_a_ ~ 9.2) and a single carboxylic acid group (pK_a_ ~ 2.5) Figure 2. These two groups have been proven to play a major role in the association process with ionizable analytes.

Under RP conditions, ionic and H-bond interactions as well as hydrophobic inclusion complexations are usually at the basis of the chiral recognition mechanism [36,37,38,39]. For ionizable compounds, the first dominating interaction involves either the carboxyl or the amino group of the chiral selector (depending on the chemical characteristics of the analyte). Then, the other directional interactions initiate the enantioselectivity.

Encouraged by the profitable results obtained by Berthod and co-workers with environmentally sustainable water-MeOH (40:60, *v/v*) mobile phases, we firstly evaluated the unprecedented possibility to replace MeOH with an equal volume of the less toxic EtOH, which was the same organic modifier used for the achiral analyses. First of all, a comparative evaluation was performed using four representative AAs contained in the investigated food supplement, namely, the charged/basic Lys, the apolar aromatic Phe, the aliphatic Val, and the polar Thr. For all analyses, the (pwsH) of the mobile phase was fixed at 3.9 with AcOH because this pH value was demonstrated as suitable for the elution of Lys which is otherwise too much strongly retained in the columns [37]. The results of the comparative evaluation are listed in Table 3.

For all four compounds, EtOH produced sensitively higher retention and *R_S_* values than MeOH. Instead, except for Phe, only a modest improvement in enantioselectivity (*α*) was obtained with the less polar alcohol. The higher solubility (which means a higher solvation power) of the tested AAs in the water-MeOH mobile phase contributed to produce shorter retention times as a result of higher attitude to remain in the bulk eluent. This retention profile together with the better elution efficiency almost always produced by EtOH (expressed as theoretical plate number, N, in Table 3) well explain the higher *R_S_* values achieved with this alcohol. Indeed, apart from Phe, only a marginal contribution to the resolution factor was given by the slightly improved enantioselectivity with EtOH. The higher elution efficiency produced by this alcohol can be mostly explained by the higher retentions, since only negligible differences in terms of peaks width were recorded with the two alcohols for the same compound (data not shown). Moreover, it is worth of mention that with EtOH, the first eluting L-enantiomer displayed always a more favorable mass transfer kinetics than its D antipode. On the contrary, a coherent trend was missing with MeOH.

According to a one-variable-at-time (OVAT) approach, the type of acid in the water-EtOH system was also evaluated. In this framework, the performance of the system was tested using FA in place of AcOH. FA is stronger than AcOH, and therefore, less needs to be added to reach the required pH. Using the same four test compounds as before, a general decline in terms of *R_S_* value was revealed with FA (Table 3). The same negative trend was also found for the N values, while (apart from Phe) enantioselectivity was nearly unaffected by this experimental modification.

In line with the retention profile observed with other zwitterion-type CSPs (*Cinchona* alkaloid-based CSPs) [40], also with the teicoplanin-based CSP, the elution strength increased with the acidity strength of the additive: FA > AcOH. This chromatographic behavior can be tentatively explained by the higher affinity of FA for the cationic site of the chiral selector.

Apart from Lys, the trend of retention mostly followed that of hydrophobicity revealing an underlying RP mechanism.

It has been recently reported with *Cinchona* alkaloid-based zwitterionic CSPs [40,41] that the use of FA and AcOH as mobile phase additives in RP and polar-ionic eluents can lead to remarkably different chromatographic performances, mostly in terms of retention profiles. Indeed, although non-chiral in nature, these two acids are capable of participating differently both in the formation and in the composition of the solvation shell surrounding the cationic moiety of the chiral selector. The higher retentions produced by AcOH over FA (Table 3) can be tentatively explained with the ability by the weaker acid to create solvation shells of smaller dimension on the chiral selector cationic function, thereby leading to stronger electrostatic attractions with the analytes [40,41]. On the other hand, the rather similar *α* values indicate that the two acidic additives did not induce different conformations of the chiral selector and analyte. This behavior highlights the lack of an acidic-specific modification in the orientation between complementary functional groups.

All these preliminary evaluations allowed us to identify the water/EtOH (40:60, *v/v*; (pwsH) 3.9 fixed with AcOH) solution as the optimal mobile phase composition to use for the purpose of this study. To the best of our knowledge, this is the first study in which this highly environmentally sustainable and MS compatible mobile phase system has been used for the enantioseparation of proteinogenic amino acids. The use of EtOH in place of the originally employed MeOH contributes to reduce the intoxication risk by the operator, in a green analytical chemistry perspective.

**Figure 3:**
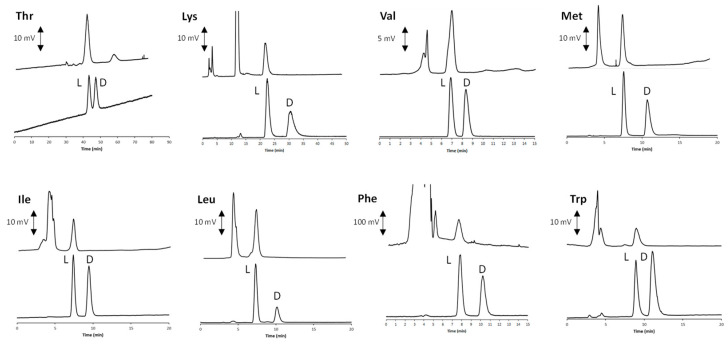
Chromatograms of all the eight AAs contained in the food supplement obtained with the optimized enantioselective analysis conditions. In each inset, bottom chromatograms refer to the analysis of the standard racemic of enantiomeric mixture, while upper chromatograms are related to the real samples after the off-line collection of the peaks from the achiral IP-RP analysis. The applied experimental conditions correspond to those obtained with mobile phase “b” in Table 3. The analysis of Thr was performed with a 0.1 mL/min flow rate, while all the remaining AAs were analyzed at 0.7 mL/min.

The optimal water-EtOH eluent was then employed for the enantioselective analysis of the remaining compounds. According to the data listed in Table 3, excellent performances were always obtained with the above RP mobile phase. The enantiomeric L < D elution order (EEO) was established by injecting pure standards of known stereochemistry. In Figure 3, the chromatograms of all the eight AAs contained in the food supplement (here analyzed as either racemates or enantiomeric mixtures) are shown (bottom lines of all inserts).

In order to assess the lowest accurately measured enantiomeric excess (*ee*) value, scalemic solutions with decreased concentrations of D-Leu and D-Phe were consecutively analyzed with the optimized enantioselective LC conditions. As a result, *ee* values ≤ 97% and 98% (both in favor of the L-enantiomer) can be accurately measured for Leu and Phe, respectively.

Once identified, the highly environmentally sustainable best conditions for the enantioselective analysis were applied to the real sample. Two consecutive injections performed with the previously optimized achiral chromatography method were enough to get suitable amounts of the peaks to re-inject into the enantioselective system. More in detail, after the off-line collections with the achiral system, the solutions were evaporated to dryness under a stream of nitrogen and the residue dissolved in 30 μL of the water-EtOH mobile phase. In order to improve the detection sensitivity, an evaporative light scattering detector (ELSD) was used for the analysis of aliphatic compounds. Instead, the analysis of Phe was followed with a conventional spectrophotometric UV–VIS detector, while a spectrofluorimetric detector was used for Trp. Details about the detection settings are reported in (pwsH) 2.3. As a result of these analyses, according to the manufacturer’s declaration, only L-enantiomers were detected (upper lines of all the inserts).

The chromatogram of Lys in Figure 3 was obtained with a slightly modified eluent system in which AcOH was replaced by 45 mM ammonium acetate, while keeping unaltered the (pwsH). This modification of the original method remarkably improved both the thermodynamic and the kinetic features of the chromatographic process. Indeed, excessive retention and poor peak shape were obtained when the sole AcOH was used as additive (Figure S2). The profitable incorporation of the buffer system reduced the retention of both Lys enantiomers by almost two-times, while contemporarily enhanced the resolution factor values (2.92), while keeping almost unaltered the *α* value (1.40). This finding clearly indicates the outstanding role played by the carboxylic group of the chiral selector in the analyte retention mechanism.

**Figure 4:**
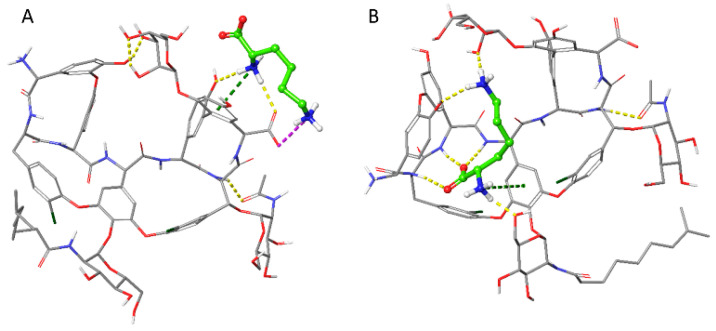
Best interaction frames of the molecular dynamics run of the teicoplanin with the Lys, in grey and green carbon atoms, respectively. The hydrogen bond, π-cation, and charge–charge interactions are shown in yellow, green, and magenta dashed lines, respectively. The panel (**A**) displays the frame where the Lys engages multiple interactions with the carboxylic moiety of the selector, while panel (**B**) shows the interactions engaged in the teicoplanin basket.

At (pwsH) 3.9, both the analyte and the chiral selector display zwitterionic characteristics. In this scenario, ammonium ions from the buffer system behave as competing species towards the anionic site (the carboxylate moiety) of the chiral selector units, thus contributing to improve the thermodynamics and, mostly, the kinetics of the enantioseparation process. In silico studies through MD simulations confirmed the simultaneous interaction between both Lys amino groups and the anionic moiety of the chiral selector, through a H-bond supporting two charge–charge interactions (Figure 4A). The positively charged moieties of the Lys have been found also to engage *p*-cation interactions with aromatic rings when the complex is formed that further stabilize the multiple hydrogen bonds formed by the analyte in the teicoplanin aglycone basket (Figure 4B). Interestingly, the two selector–selectand associates reported are the most stable frames of the whole trajectory, characterized by an energy difference of −133.7 kcal/mol for the former and −134 kcal/mol for the latter.

As a proof-of-concept, a simple and easily feasible 2D-HPLC system was implemented to demonstrate the possibility to speed up the comprehensive analysis of the food supplement. As a test compound, *rac*-Phe was selected for its favorable spectroscopic characteristics that make possible the use of UV–VIS detectors in the two connected HPLC systems.

The analytical platform used in this study was characterized by three interconnected units, according to the so-called “heart-cut” method [42,43]: (1) the achiral RP system employed for the separation of the AAs in the first dimension; (2) the two-position six-port switching valve enabling the connection of the achiral system with (3) the second dimension chiral system in which Phe peak from the first (achiral) column was enantioseparated with the teicoplanin-based CSP.

The chromatogram in Figure S3 clearly shows that this approach can be applied in principle and that the HFBA used in the first achiral method does not disturb the enantioseparation process in the second dimension. The reduced chromatographic performance readily evident in the chiral analysis of *rac*-Phe with the 2D system can be imputed to the necessity to improve the transfer from one apparatus to another, which was not the scope of the present study.

## 3. Materials and Methods

### 3.1. Chemicals and Reagents

Analytical grade methanol (MeOH), ethanol (EtOH), acetic acid (AcOH), formic acid (FA), ammonium acetate, heptafluorobutyric acid (HFBA), and all the eight amino acid standards (AAs) were purchased from Merck Life Science (Merck KGaA, Darmstadt, Germany). A commercial original dietary supplement labelled to contain only L-AAs was obtained from a local drug store. Water for HPLC analysis was purified with a New Human Power I Scholar water purification system (Human Corporation, Seoul, Korea) and Milli-Q water purification system from Millipore (Milan, Italy). Mobile phases were degassed with 10 min sonication before use. The “apparent” (pwsH), that is, the one measured in the employed hydro-organic mobile phase (s), while the calibration of the pH system was carried out in water (w) of the eluent, was measured with a conventional pH-meter (Hanna Instruments™, Woonsocket, Rhode Island, USA) and then opportunely adjusted to the required value with FA or AcOH.

### 3.2. Extraction of the AA Pool from the Food Supplement

Five sachets of oral granules (the commercial food supplement) were weighted and accurately grounded in a mortar to obtain a homogeneous fine powder. About 200 mg of powder were weighted, transferred into a volumetric flask, and solubilized with a water/EtOH (95:5, *v/v*) solution to get a final 1.5 mg/mL concentration. The resulting suspension was sonicated 5 min to facilitate the extraction of all eight AAs, and then, 5 mL were filtered through a 0.45 µm nylon filter. The filtered solution was used for the achiral IP-RP-HPLC analysis as such.

### 3.3. Calibration Curves for Quantitative Analysis

After some preliminary tests, a 1.5 mg/mL concentration of food supplement was found to be optimal for the quantitative analysis. At this concentration, the peaks of all eight AAs extracted from the food supplement were clearly distinguishable from the baseline without any detector saturation. In Section 3.2 more details about the sample preparation are reported. For each AA, the quantitative analysis was performed using calibration curves properly constructed by plotting the concentration of five calibration standard solutions against the measured response (peak area value) (Table S1, Supporting Information). For all compounds, the area value of the peak measured during the analysis of the real sample was comprised in the range of the area values obtained analyzing five selected calibration standard solutions. Additionally, each calibration curve was built up by taking into account the AA content declared by the producer. In this respect, a range of concentration between 50–150% of the theoretical AA concentration was used. The extremely high R^2^ values (>0.999) obtained for all compounds indicated the high linearity of the obtained mathematical models.

### 3.4. Achiral and Chiral HPLC Analysis

The analytical HPLC measurements were made on a Shimadzu (Kyoto, Japan) LC-20A Prominence, equipped with a CBM-20A communication bus module, two LC-20AD dual piston pumps, an SPD-M20A photodiode array detector, an RF-10A fluorescence detector, and a Rheodyne 7725i injector (Rheodyne Inc., Cotati, CA, USA) with a 20 µL stainless steel loop. The Prevail RP18 column (Grace, Sedriano, Italy, 250 mm × 4.6 mm i.d., 5 μm, 110Å pore size) was used as the analytical column for all the achiral ion pairing IP-RP-HPLC analysis. The IP-RP-HPLC method was optimized at both 1.0 mL/min and 1.2 mL/min flow rates. The gradient profile at a flow rate of 1.0 mL/min was run with water/HFBA (99:0.1, *v/v*; eluent A) and plain EtOH (eluent B) under the following elution program: 0–37 min, 95% A; 37–70, 70% A. The gradient profile, setting the flow rate at 1.2 mL/min, was run with water/HFBA (99:0.1, *v/v*; eluent A) and plain EtOH (eluent B) under the following elution program: 0–27 min, 95% A; 27–50, 70% A. The chromatograms were followed with an UV–VIS detector at 220 nm.

For the enantioselective chromatography analysis, a Chirobiotic T column (250 mm × 4.6 mm I.D., containing the glycopeptide Teicoplanin A2-2 covalently bonded to a high purity 5 µm spherical silica gel) from Sigma-Aldrich (Milano, Italy) was used. Before use, the column was conditioned with 40 mL of the selected eluent system. Column temperature was fixed at 25 °C with a Grace (Sedriano, Italy) heater/chiller (Model 7956R) thermostat. Chromatograms were obtained and handled with the LC Solution Software from Shimadzu (Kyoto, Japan). The analysis of Ile, Leu, Lys, Met, Thr, and Val was monitored with the evaporative light scattering detector (ELSD) Sedex 55 (S.E.D.E.RE., France) operated with the following setting: drift tube temperature operating, 40 °C; nitrogen pressure, 240 kPa. The analog-to-digital conversion of the output signal from the ELSD was allowed by a common interface device. The analysis of Phe was monitored at 254 nm with a conventional UV–VIS detector, while that of Trp with a fluorescence detector setting λ_ex_ 270 nm and λ_em_ at 350 nm. All the analyses were performed at a 0.7 mL/min flow rate.

For the 2D-HPLC system, the enantioselective analysis was performed on a Shimadzu (Kyoto, Japan) LC-Workstation Class LC-10 equipped with a CBM-10A system controller, two LC-10AD high pressure binary gradient delivery systems, and an SPD-10A variable-wavelength UV–VIS detector. The two-position six-port switching valve including a 100 μL stainless steel loop was assembled in our laboratories using a conventional Rheodyne injector.

### 3.5. Computational Methods

The Maestro graphical interface of the Schrödinger Suite 2021-3 (Schrödinger, LLC, New York, NY, USA, 2021) was used. The three-dimensional (3D) structure of the chiral selector Teicoplanin A2-2 was retrieved from the Protein Data Bank (PDB code 4PJZ) [44] and checked for missing atoms, bonds, and stereochemistry correctness. Lysine and Teicoplanin A2-2 contain multiple ionizable groups; thus, the MoKa 2.6.6 software [45] was used to correctly assign the predominant tautomeric and protomeric forms. An orthorhombic box was then built with a 41.7 Å side length and filled by 1340 molecules of water/EtOH, a custom solvent reproducing a 60:40 (*v/v*) mixture. This stage was performed with the aid of a Packmol tool [46] leading to the generation of the solvated simulation systems.

The molecular dynamics were conducted in the ensemble class NVT with a temperature in the simulation cell of 298K kept constant with a Nosé–Hoover thermostat [47,48]. All the other parameters in the analysis were set to default values in the Desmond molecular dynamics package of the Schrödinger Suite 2021-3 [49], which means, among others, a 2 fs integration timestep and the OPLS4 as force field [50]. Next, a production run produced 1000 frames during the 1 µs dynamics (1000 ps recording interval). In order to support our interaction hypothesis at a molecular level, the attention was focused on monitoring the charge–charge interactions occurring between the positively charged amino groups of the lysine and the negatively charged carboxylic group of the Teicoplanin-A2-2, together with the selector–selectand interaction energies.

## 4. Conclusions

In this work, we have demonstrated for the first time that a valuable performance can be obtained replacing ACN with EtOH as organic modifier in the direct IP-RP-HPLC analysis of AAs without compromising the statistical quality of the chromatographic method. The data reported in this manuscript further confirm that very low concentrations of HFBA (0.1%, *v/v*) can be conveniently used for the selective and efficient analysis of a heterogeneous pool of AAs both under both isocratic and gradient condition, without the need for pre-analysis derivatization steps.

In the present work, we have also firstly demonstrated that RP eluents based on water-EtOH mixtures with low amounts of AcOH (or ammonium acetate) produce excellent direct enantioseparations of underivatized AAs (with α and R_S_ values up to 4.6 and 15.0, respectively).

Very profitably, the environmentally sustainable and safe achiral and chiral methods developed in this study can be used for the overall analysis of a food supplement containing essential AAs. The use of a fully volatile acidic additive in the IP-RP-HPLC achiral system makes possible the easy off-line enantioselective analysis of all the eight AAs of the investigated pool with different detection systems. As a proof-of-concept, we have also demonstrated that the two methods can be conveniently combined in a 2D-HPLC configuration to perform chemoselective and enantioselective investigations through the “heart-cutting” approach.

As far as the quantitative analysis of the selected food supplement is concerned, the developed methods have revealed that (i) the content of six out of eight AAs is consistent with the producer declaration, the lower amount of Met and Trp being tentatively ascribed to oxidative degradation, and (ii) all AAs are present exclusively as L enantiomers.

## Figures and Tables

**Table 1:** Chromatographic performance obtained with the tested experimental conditions (see the footnote for details). *k*: Retention factor; *α*: separation factor; *R_S_*: resolution factor. All the analyses were followed at 220 nm. Separation (*α*) and resolution (*R_S_*) factor values are referred to two consecutive peaks.

**Compound**	**Mobile Phase Composition**								
	**a**			**b**			**c**		
	***k***	***α***	***R_s_***	***k***	***α***	***R_s_***	***k***	***α***	***R_s_***
Thr	0.62			0.51			0.60		
Lys	1.71	2.75	6.39	1.21	2.35	4.61	2.07	3.46	8.59
Val	1.71	1.00	N.R.	1.46	1.21	1.22	2.38	1.15	1.50
Met	2.73	1.60	3.63	2.20	1.51	3.31	2.68	1.13	1.81
Ile	4.19	1.53	3.94	3.10	1.41	3.76	5.36	2.00	9.85
Leu	5.01	1.20	1.71	3.64	1.18	1.99	6.35	1.18	2.52
Phe	6.07	1.21	1.68	4.32	1.19	2.20	8.71	1.37	5.45
Trp	10.98	1.81	4.86	11.21	2.59	9.43	25.06	2.88	17.71
**Compound**	**Mobile Phase Composition**								
	**d**			**e**			**f**		
	***k***	***α***	***R_s_***	***k***	***α***	***R_s_***	***k***	***α***	***R_s_***
Thr	0.48			0.48			0.44		
Lys	2.07	4.29	14.66	2.07	4.29	14.66	2.01	4.60	14.95
Val	2.54	1.23	4.22	2.54	1.23	4.22	2.51	1.25	4.10
Met	2.99	1.18	3.60	2.99	1.18	3.60	2.86	1.14	2.45
Ile	7.91	2.64	22.52	7.91	2.64	22.52	6.80	2.38	9.56
Leu	8.97	1.13	3.32	8.97	1.13	3.32	8.02	1.18	2.01
Phe	15.56	1.77	18.27	15.56	1.13	18.27	13.27	1.65	10.01
Trp	N.E.			18.92	1.35	26.98	20.13	1.53	15.04

a: water/EtOH/HFBA (80:20:0.2, *v/v/v*). b: water/EtOH/HFBA (80:20:0.1, *v/v/v*). c: water/EtOH/HFBA (90:10:0.1, *v/v/v*). d: water/EtOH/HFBA (95:5:0.1, *v/v/v*). e: water/HFBA (99:0.1, *v/v*; eluent A) and plain EtOH (eluent B); gradient program: 0–37 min, 95% A; 37–70, 70% A; flow rate 1.0 mL/min. f: water/HFBA (99:0.1, *v/v*; eluent A) and plain EtOH (eluent B); gradient program: 0–27 min, 95% A; 27–50 min, 70% A; flow rate 1.2 mL/min. N.E.: not eluted.

**Table 2:** Method validation: evaluation of precision (RSD%) and accuracy (Recovery%) in the short- and log-term period (intra-day and inter-day precision and accuracy values) for Leu and Phe.

Compound	Theoretical Conc. (mg/mL)	Intra-Day Mean Conc. (mg/mL)	Intra-Day Precision (RSD%)	Intra-Day Accuracy (Recovery%)	Inter-Day Mean Conc. (mg/mL)	Inter-Day Precision (RSD%)	Inter-Day Accuracy (Recovery%)
Leu	0.11	0.11	1.29	99.39	0.11	1.87	100.11
		0.11	1.32	99.44			
		0.11	2.33	101.50			
	0.45	0.45	0.96	99.69	0.45	1.53	99.61
		0.44	2.45	98.75			
		0.45	0.66	100.39			
Phe	0.02	0.02	1.82	99.08	0.02	1.77	98.31
		0.02	1.97	96.77			
		0.02	1.04	99.07			
	0.16	0.16	1.78	99.51	0.16	1.48	100.62
		0.16	0.72	101.09			
		0.16	1.58	101.26			

Intra-day and Inter-day evaluation: analysis of 3 replicates of each external set within one day and for three consecutive days.

**Table 3:** Chromatographic performance obtained with the tested experimental conditions (see the footnote for details). *k*_2_: Retention factor of the second eluted D-enantiomer of each pair; *α*: separation factor; *N*_2_: number of theoretical plates of the second eluted enantiomer of each pair; *R_S_*: resolution factor. All the analyses were followed at 220 nm.

Mobile Phase	Compound	*k*_2_^d^	*α*	*N*_2_^d^	*R_S_*
a	Lys	7.66	1.31	912	1.58
	Phe	1.55	1.29	2285	1.79
	Thr	0.34	1.39	2681	0.99
	Val	0.64	1.48	1316	1.44
b	Lys	12.9	1.32	1437	1.80
	Phe	1.57	1.77	3565	4.81
	Thr	0.52	1.44	4765	1.95
	Val	0.95	1.59	2302	2.78
	Ile	1.28	1.63	2849	3.31
	Leu	1.5	1.94	3603	5.28
	Met	1.45	2.16	1681	4.24
	Trp	1.68	1.74	3193	4.66
c	Lys	4.32	1.39	722	1.54
	Phe	1.49	1.31	2118	1.84
	Thr	0.4	1.4	N.C. ^e^	N.C. ^e^
	Val	0.79	1.53	699	1.39

a: water/MeOH (60:40, *v/v*), apparent pH fixed at 3.9 with AcOH. b: water/EtOH (60:40, *v/v*), apparent pH fixed at 3.9 with AcOH. c: water/EtOH (60:40, *v/v*), apparent pH fixed at 3.9 with FA. ^d^ The subscript refers to the second eluted enantiomer of each pair. ^e^ N.C.: not calculated by the software.

## Data Availability

Not applicable.

## Supplementary Materials

The following supporting information can be downloaded at: https://www.mdpi.com/article/10.3390/molecules27227724/s1, Figure S1: Chromatogram of the mixture of eight AA standards obtained with a RP column of smaller dimensions; Figure S2: Enantioseparation of Lys with AcOH as eluent additive; Figure S3: Enantioselective analysis of *rac*-Phe obtained with a “heart-cut” 2D-HPLC configuration; Table S1: Calibration data for each selected AA.

## Sample Availability

Samples of the compounds are available from the authors.
