# Supplementary material for: Environmentally Sustainable Achiral and Chiral Chromatographic Analysis of Amino Acids in Food Supplements
Source: Molecules. 2022 Nov 9;27(22):7724. doi: 10.3390/molecules27227724 (PMC9697624; doi:10.3390/molecules27227724)
Supplement: Supplementary file 1 [file molecules-27-07724-s001.zip › molecules-1993055-supplementary.pdf]

Supplementary Information

Environmentally sustainable achiral and chiral chromatographic analysis of amino acids in food supplements

Ina Varfaj[a,ξ], Andrea Carotti[a,ξ], Luciano Mangiapelo[a], Lina Cossignani[a,b], Agnese Taticchi[c], Antonio Macchiarulo[a], Federica Ianni[a]*, Roccaldo Sardella[a,b]

[a] Department of Pharmaceutical Sciences, University of Perugia, Via Fabretti 48, 06123 Perugia (Italy)

[b] Center for Perinatal and Reproductive Medicine, University of Perugia, Santa Maria della Misericordia University Hospital, 06132 Perugia (Italy)

[c] Department of Agricultural Food and Environmental Sciences, University of Perugia, Via S. Costanzo, 06126 Perugia (Italy)

*Correspondence to: federica.ianni@unipg.it

[ξ]The two authors contributed equally to this paper.

HPLC-DAD analysis with the Waters Xterra MS C18 column

The HPLC-DAD analysis was carried out using the following conditions: column, Waters XTerra MS C18 (150 x 4.6 mm; 5 µm particle size; 125 Å pore size); mobile phase, eluent A (0.1% v/v HFBA in water) and eluent B (EtOH) [gradient program: 0-13.5 min, 95% A; 13.5-18.5 min, from 95% to 70% A; 18.5-30 min the eluent is kept constant to 70% A; 30.1-50 min 95% A]; flow rate, 1.0 mL/min; column temperature, 25 °C; the analysis was monitored at 220 for the aliphatic AAs while at 254 nm for the two aromatic ones.
The HPLC-DAD study was performed on a Waters ALLIANCE 2695 Separations Module system equipped with a quaternary, low-pressure mixing pump and in-line vacuum degassing, an autosampler with maximum capacity of 120 vials and a column heather/cooler. The system is endowed with a photodiode array detector (Waters 2996). The data management was made by means of a Waters® Millennium®32 Software.

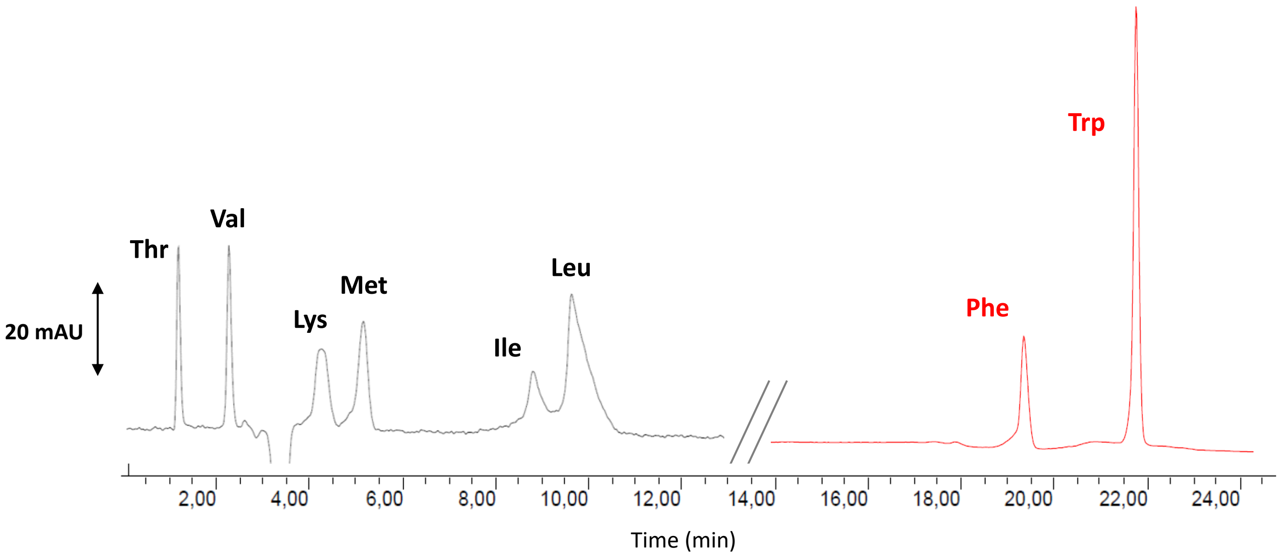

Figure S1. Chromatogram of the mixture of eight AA standards obtained with a RP column of smaller dimensions.

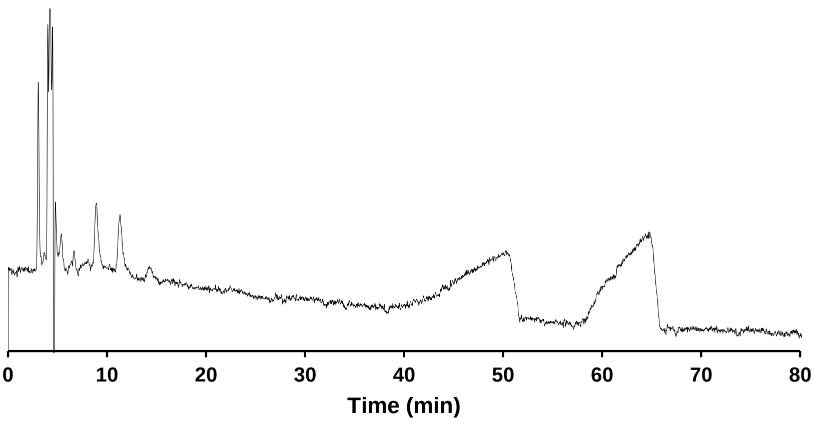

Figure S2. Enantioseparation of Lys under the following experimental conditions: column, teicoplanin-based CSP; eluent, water-EtOH (60:40, v/v), ${}_{w}^{s}pH$ fixed at 3.9 with AcOH; column temperature, 25 °C; flow rate, 0.7 mL/min; UV-Vis detector set at 220 nm.

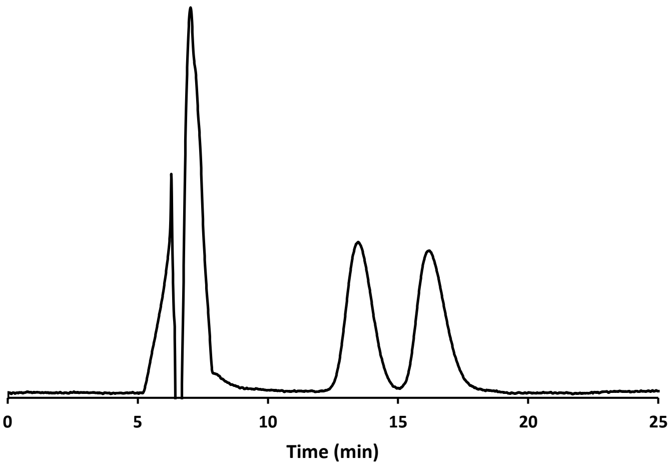

Figure S3. Enantioselective analysis of *rac*-Phe obtained with the 2D-HPLC configuration according to the "heart-cut" approach and under the following experimental conditions: column, teicoplanin-based CSP; eluent, water/EtOH (60:40, v/v), ${}^{s}_{w}pH$ fixed at 3.9 with AcOH; column temperature, 25 °C; flow rate, 0.7 mL/min; UV-Vis detector set at 254 nm.

Table S1. Linearity concentration (mg/mL) range for each selected amino acid (AA). In all the cases, correlation coefficient values (R^2) higher than 0.999 were measured.

Compound	Linearity Conc. Range (mg/mL)
Thr	0.13-2.00
Lys	0.03-0.52
Val	0.08-1.25
Met	0.03-0.50
Ile	0.15-2.50
Leu	0.05-0.55
Phe	0.02-0.20
Trp	0.01-0.20
